# Network approach reveals the spatiotemporal influence of traffic on air pollution under COVID-19

**DOI:** 10.1063/5.0087844

**Published:** 2022-04-26

**Authors:** Weiping Wang, Saini Yang, Kai Yin, Zhidan Zhao, Na Ying, Jingfang Fan

**Affiliations:** 1 School of National Safety and Emergency Management, Beijing Normal University, Zhuhai 519087, China; 2 State Key Laboratory of Earth Surface Processes and Resources Ecology, Beijing Normal University, Beijing 100875, China; 3 School of Traffic and Transportation, Beijing Jiaotong University, Beijing 100044, China; 4 China Complexity Computation Lab, Department of Computer Science, School of Engineering, Shantou University, Shantou 515063, China; 5 China State Key Laboratory of Environmental Criteria and Risk Assessment, Chinese Research Academy of Environmental Sciences, Beijing 100012, China; 6 School of Systems Science, Beijing Normal University, Beijing 100875, China

## Abstract

Air pollution causes widespread environmental and health problems and severely hinders the quality of life of urban residents. Traffic is critical for human life, but its emissions are a major source of pollution, aggravating urban air pollution. However, the complex interaction between traffic emissions and air pollution in cities and regions has not yet been revealed. In particular, the spread of COVID-19 has led various cities and regions to implement different traffic restriction policies according to the local epidemic situation, which provides the possibility to explore the relationship between urban traffic and air pollution. Here, we explore the influence of traffic on air pollution by reconstructing a multi-layer complex network base on the traffic index and air quality index. We uncover that air quality in the Beijing–Tianjin–Hebei (BTH), Chengdu–Chongqing Economic Circle (CCS), and Central China (CC) regions is significantly influenced by the surrounding traffic conditions after the outbreak. Under different stages of the fight against the epidemic, the influence of traffic in some regions on air pollution reaches the maximum in stage 2 (also called Initial Progress in Containing the Virus). For the BTH and CC regions, the impact of traffic on air quality becomes bigger in the first two stages and then decreases, while for CC, a significant impact occurs in phase 3 among the other regions. For other regions in the country, however, the changes are not evident. Our presented network-based framework provides a new perspective in the field of transportation and environment and may be helpful in guiding the government to formulate air pollution mitigation and traffic restriction policies.

Increasing air pollution has a significant negative impact on human health. Urban traffic emissions will increase air pollution. However, their dynamic modes among cities and regions remain a significant challenge. The impact of COVID-19 has forced cities and regions to implement different traffic restriction policies one after another, which naturally becomes a controlled experiment to reveal their relationship. In the present work, we develop a multi-layer network-based framework with the traffic index and air quality index. We find that air quality is related to their surrounding traffic conditions in the BTH, CCS, and CC regions when the epidemic spreads. In addition, to study the influence of traffic on air pollution, different stages in the fight against the epidemic are identified. Our method and results presented here not only provide a deep understanding of the influence of traffic on air pollution but can also be applied to study other climate and environment phenomena such as global warming.

## I. INTRODUCTION

With the use of fossil energy sources such as transportation, industry, agriculture, and power and the continuous increase in the daily cooking and heating needs of people, there is an eventual serious increase in air pollution. Air pollution poses a major threat to human health and can cause stroke, heart disease, lung cancer, and acute and chronic respiratory diseases.^1^ According to WHO estimates, about 91% of the world's population lives in places with poor air quality, causing about 4.2 × 10^6^ deaths per annum.^2^ The transportation system is one type of critical lifeline and is essential to the functioning of modern society.^3^ Traffic emission is a major source of air pollution in urban areas,^4,5^ and this pollution occurs as a result of carbon monoxide (CO), carbon dioxide (CO_2_), volatile organic compounds (VOCs) or hydrocarbons (HCs), nitrogen oxides (NOx), secondary aerosols formed through physical and chemical processes, and pollutant-suspended particles caused by brakes, tire wear, and rewear.^6^ Urban traffic congestion has increased traffic emissions, further increasing air pollution. Thus, it is vital to explore the relationship between traffic and air pollution.

Considerable research has been devoted to exploring the influence of traffic on air pollution. The most intuitive method is by making spatiotemporal variations of air pollutants, including CO, CO_2_, PM_2.5_, PM_10_, SO_2_, O_3_, and NO_x_. With diurnal analysis of hourly PM_2.5_, PM_10_, NO_2_, and CO concentrations, two ascending stages caused by two traffic peaks have been found.^7^ An increase in secondary organics (NH_4_)_2_SO_4_ and NH_4_NO_3_ caused by vehicle emission has also been found.^8^ Then, some statistical methods are used in this field. Multivariate autoregressive models are used to estimate pollution levels under different traffic conditions.^9^ Further, some physical approaches are proposed based on a parameterized analytical representation of the entire fuel consumption and emission process. Approaches like CMEM^10^ and MOBILE^11^ can simulate and estimate CO, HC, and NOx emitted by traffic.^12^ Experimental studies are also exploring the impact of traffic and its emissions on air pollution. Combining the chassis dynamometer system and an outdoor enclosed environmental chamber, new particle formation from traffic emissions has been assessed. The new particle formation can frequently produce high levels of ultrafine particles, causing serious air pollution.^13^ However, the above methods are either microscopically unable to explore the relationship between traffic and air pollution in large-scale areas (like physical and experimental methods) or generalize and fail to reveal the mechanism of how regional traffic variability causes a change in air pollution (like statistical methods).

With the coronavirus pandemic sweeping the world, many countries have implemented strict lockdown policies to stop the spread of the disease. These policies, especially limited transportation activities, will improve ambient air quality. This has been confirmed in some cities or regions in China,^4–6,14–24^ Egypt,^25^ Spain,^26^ France,^26^ Italy,^26–28^ Brazil,^29–31^ Korea,^32^ New Zealand,^33^ Singapore,^34^ the United States,^9,26,35^ Malaysia,^36^ East Asia,^37^ Europe,^38^ and even at the global level.^39^ However, a study^40^ has also found that the overall air quality in urban areas in China has not improved even with the COVID-19 lockdown. Besides, as the world gradually opens up every time a wave ends, there is relaxation in movement restrictions, paving the way for a return of harsh air in some city in Asia. For example, India is worse off now than before.^41^ With the lifting of the lockdown in some areas of China, large-scale movement of people and goods began, and air pollution has gradually returned to, or is likely to exceed, the levels before the lockdown.^42^ Therefore, with the different development stages and response measures of the pandemic, the contribution of traffic to air pollution will also change significantly. An improved understanding of the role of traffic in air pollution during the spatiotemporal changes occurring in COVID-19 is, thus, needed, since it is beneficial to reduce air emissions through regulation and incentives.

In past years, network theory has been found useful for better understanding spatiotemporal behavior in the climate system.^43–48^ Climate networks establish correlations among climate anomalies in distant parts of the world and attempt to explain them using relevant physical progress. In a climate network, geography data are transformed into nodes and edges of a network that can represent spatiotemporal relationships. Nodes refer to geographical locations or grid sites, and edges are constructed based on similarities (such as cross correlations) in the variability over time between pairs of nodes. Various climate data records (such as temperature, pressure, winds, and precipitation) can be used to construct a climate network. The climate network approach can provide a powerful framework to better understand the structure and pattern of climate phenomena, including air pollution.^43^

The basic idea behind climate networks is that relevant and important features of atmospheric mechanisms influence the variability of the traffic index to air pollution at different locations, and these influences are encoded in the structure of the network. By extracting the topological index of the network, we can reveal the underlying links from traffic to air pollution. In this study, we develop a network-based framework to explore the influence of traffic on air pollution during the temporal and spatial changes in COVID-19. A multi-layer network between traffic index and air pollution is reconstructed. Our results can help formulate strategies and countermeasures for traffic emission and air pollution.

## II. DATA

### A. Traffic index data

In this study, we collect the traffic index data from TOMTOM (https://www.tomtom.com/en_gb/traffic-index/). These data can represent congestion levels in Chinese cities. Here, we employ the daily traffic index (TL) of 21 major cities from January 1 to July for 2019 and 2020, respectively. The TL is measured by calculating the proportion of increase in the actual travel time over free flow travel time, and its value is greater than or equal to 0. The larger the indicator value, the more severe is the traffic congestion.

### B. Air quality index

Daily air quality index (AQI) data acquired from the China National Environmental Monitoring Centre (CNEMC) are used in this study. The AQI is based on ambient air quality standards and the impact of various pollutants on human health, ecology, and the environment and simplifies the concentrations of several air pollutants that are routinely monitored into a single index value. The value range of the AQI is set from 0 to 500. The larger the value, the more serious the air pollution. According to traffic index (TL) records, the AQI of 21 major cities for the period between January 1 and July for the years 2019 and 2020 is selected.

## III. METHODS

### A. Data pre-processing

In this study, we divided 21 cities into six region groups according to the existing geographical divisions, namely, Beijing–Tianjin–Hebei (BTH), Northeast China (NEC), the Chengdu–Chongqing Economic Circle (CCS), Central China (CC), the Guangdong–Hong Kong–Macao Greater Bay Area (GHM), and the Yangtze River Delta (YRD) as shown in Table I. BTH includes two municipalities, Beijing and Tianjin, and is located at the heart of the Bohai Rim in China. It is the largest and most dynamic region in northern China, one of the regions with the greatest potential for economic development in China, and one of the regions with the most extensive transportation and logistics network. A one-hour traffic circle with rail transit was initially formed. NEC is the general term for land in the northeast of China. The economy of NEC started early and has made great historical contributions to the development and growth of China. However, due to the severe loss of youth and the impact of cold weather, the economy of NEC has failed to keep pace with that of the rest of the country in the past 30 years, thus also affecting the building of transportation infrastructure. The CCS is an urbanized area with the highest level of development and great development potential in western China. It is an important part of the implementation of the Yangtze River Economic Belt and the Belt and Road strategy. It is the starting point of the new land-sea corridor in the west and has the unique advantage of linking the southwest and northwest and connecting East Asia with Southeast Asia and South Asia. The CCC is located in the central part of China, with many national transportation trunk lines reaching the whole country. It has the advantage of being a strategic hub in the east, west, north, and south of the country and a water and land transportation hub. Economically, it is considered to be a relatively underdeveloped area. The GHM includes Hong Kong, Macau, Guangzhou, Shenzhen, and other cities. The GHM, the New York Bay Area, the San Francisco Bay Area, and the Tokyo Bay Area of Japan are also known as the four major bay areas in the world. The GHM is one of the regions with the highest degree of openness and the strongest economic vitality in China. It occupies an important strategic position in the overall development of the country, paving the way for the formation of a convenient, efficient, modern, and comprehensive transportation system. The YRD is an important intersection between the “Belt and Road Initiative” and the Yangtze River Economic Belt. It is an important platform for China to participate in international competition, an important engine for economic and social development, and one of the regions with the best urban foundation in China. In terms of the density of highway and railway transportation lines, the YRD leads the country, paving the way for the formation of a three-dimensional comprehensive transportation network.

**TABLE I:** Outbreak level for COVID-19 among cities.

City	Outbreak level	Region group	Cumulative confirmed cases
Beijing	4	BTH	1 049
Tianjin	3	BTH	364
Shijiazhuang	3	BTH	898
Shenyang	1	NEC	70
Changchun	2	NEC	150
Chengdu	2	CCS	158
Chongqing	3	CCS	591
Wuhan	4	CC	50 340
Changsha	3	CC	242
Guangzhou	3	GHM	377
Shenzhen	3	GHM	423
Zhuhai	1	GHM	98
Dongguan	1	GHM	99
Xiamen	1	GHM	35
Quanzhou	1	YRD	47
Shanghai	4	YRD	1 840
Suzhou	1	YRD	87
Wuxi	1	YRD	55
Nanjing	1	YRD	93
Hangzhou	2	YRD	181
Ningbo	2	YRD	157

By using the cumulative confirmed cases of cities as of March 16, 2021, to represent the risk of COVID 19, we can classify cities into four outbreak levels: 1: [0,100), 2: [100, 300), 3: [300, 1000), and 4: [1000, +∞) as shown in Table I. The higher the outbreak level in the cities, the higher the number of infected people and the wider the spread of the epidemic. Here, the effect of seasonality on the AQI has been removed by subtracting the calendar day's mean from the original datasets.

### B. Network construction

Similar to earlier studies,^44,45^ we define the $X_{T_{j,}A_{i}}(\tau )$ as the time-delayed cross-correlational function for the TL node *j* and AQI node *i*, denoted by $X_{T_{j},A_{i}}(\tau )$: for *τ *≥ 0,

$$X_{T_{j},\;A_{i}}(\tau )=\frac{\sum_{t=1}^{L-\tau }(A_{i}(t)-{\bar{A\;}\;}_{i})(T_{j}(t+\tau )-{\bar{T\;}\;}_{j})}{\sqrt{\sum_{t=1}^{L-\tau }{\left(A_{i}(t)-{\bar{A\;}\;}_{i}\right)}^{2}\cdot }\sqrt{\sum_{t=1}^{L-\tau }{\left(T_{j}(t+\tau )-{\bar{T\;}\;}_{j}\right)}^{2}}},$$ (1)

and for *τ* < 0,

$$\begin{array}{r} X_{T_{j},A_{i}}(-\tau )=\frac{\sum_{t=1}^{L-\tau }(A_{i}(t+\tau )-{\bar{A\;}\;}_{i})(T_{j}(t)-{\bar{T\;}\;}_{j})}{\sqrt{\sum_{t=1}^{L-\tau }{\left(A_{i}(t+\tau )-{\bar{A\;}\;}_{i}\right)}^{2}\cdot }\sqrt{\sum_{t=1}^{L-\tau }{\left(T_{j}(t)-{\bar{T\;}\;}_{j}\right)}^{2}}}, \end{array}$$ (2)

where ${\bar{A\;}\;}_{i}$ and ${\bar{T\;}\;}_{j}$ denotes the average of AQI time series and TL time series. The time lags τ are in the range of −7 and +7 days. The time lag is chosen to be long enough to avoid the sensitive of correlation estimation to our choice of time lag, which leads to erroneous correlation estimation. The deviations in the link identification due to persistence or autocorrelation in the records are reduced by dividing the $std(X_{j,i})$. The strength of the positive and negative link weights is denoted as

$$W_{T_{j},\;A_{i}}^{pos}=\frac{\left(\max(X_{T_{j},A_{i}})-mean(X_{T_{j},A_{i}})\right)}{std(X_{T_{j},A_{i}})}$$ (3)

$$W_{A_{i},T_{j}}^{neg}=\frac{\left(\min(X_{T_{j},A_{i}})-mean(X_{T_{j},A_{i}})\right)}{std(X_{T_{j},A_{i}})},$$ (4)

where $\max(X_{T_{j},A_{i}})$, $\min(X_{T_{j},A_{i}})$, $mean(X_{T_{j},A_{i}})$, and $std(X_{T_{j},A_{i}})$ are the maximum, the minimum, mean, and the standard deviation of the cross-correlational function, respectively. We define $\tau_{T_{j},A_{i}}^{pos}$ and $\tau_{T_{j},A_{i}}^{neg}$ as the corresponding time lags at these two peaks. When $\tau_{T_{j},A_{i}}>0$, the links are outgoing from TI nodes pointing to AQI nodes; when $\tau_{T_{j},A_{i}}<0$, the links are pointing away from AQI nodes coming toward TI nodes. Here, links with zero-time lags are excluded. The adjacency matrix of a climate network is defined as

$$\Lambda_{T_{j},A_{i}}^{pos}=\{\begin{array}{ll} 1 & \text{if}\;{\text{W}}_{T_{j},A_{i}}^{pos}\geq Q\;\text{and}\;\tau_{T_{j},A_{i}}^{pos}>0\;,\\ 0 & \text{else,} \end{array}$$ (5)

$$\Lambda_{T_{j},A_{i}}^{neg}=\{\begin{array}{ll} 1 & \text{if}\;{\text{W}}_{T_{j},A_{i}}^{neg}\leq -Q\;\text{and}\;\tau_{T_{j},A_{i}}^{neg}>0,\\ 0 & \text{else.} \end{array}$$ (6)

Here, *Q* is a threshold for the weight links, which is determined based on the shuffling procedure.^46,47^ In the shuffled case, the order of days is permutated for each pair of TI and AQI nodes *j* and *i.*^47^ By this step, we keep all the statistical quantities of the original data but omit the physical dependencies between TI and AQI nodes. In such a case, the shuffled network represents the properties of statistical quantities and the autocorrelations of the original records, which may introduce unrealistic links. If the original link weights are significantly higher than those of the control, we regard it as a real link; otherwise, they are spurious links. Then, we obtain the desired connection between the TI and the AQI based on the adjacency matrix $\Lambda_{T_{j},A_{i}}^{pos}$ and $\Lambda_{T_{j},A_{i}}^{neg}$.

The degree is the most common application for measuring climate networks. A link that points toward a node is referred to as an in-degree link, and a link that points away from a node is considered as an out-degree link. The way in which the TI is dynamically influenced by the AQI is defined as the weighted out-degree of TI nodes, which are the total outgoing link weights from TI nodes.^48^ The response of the AQI to the TI is denoted as the weighted in-degree of AQI nodes, which are the total incoming link weights pointing toward AQI nodes.

Obviously, the outgoing links of the TI are the same as the incoming links of the AQI. Nodes that have higher values represent a higher connection with other nodes in the network, while lower values mean “isolated” in the network. The in and out fields describe the level of TI nodes impacting the AQI nodes and the level of the affected AQI nodes from TI nodes, respectively.

### C. Significance tests

The statistical significance of link weights is determined based on a shuffling procedure. In the shuffled case, the order of years is permutated and the order of days within each year is maintained for each pair of TI and AQI nodes *i* and *j*.^41^ We generate shuffled data according to the procedures described in Sec. III B. This shuffling keeps all the statistical quantities of the original data but omits the physical dependencies between TI and AQI nodes. In such a case, the shuffled network represents the properties of statistical quantities and the autocorrelations of the original records, which may introduce unrealistic links. We choose a control for the records to distinguish realistic links from unrealistic ones. If the original link weights are significantly higher than those of the control, we regard them as real links; otherwise, they are spurious links.

### D. Analysis framework

**FIG. 1.**
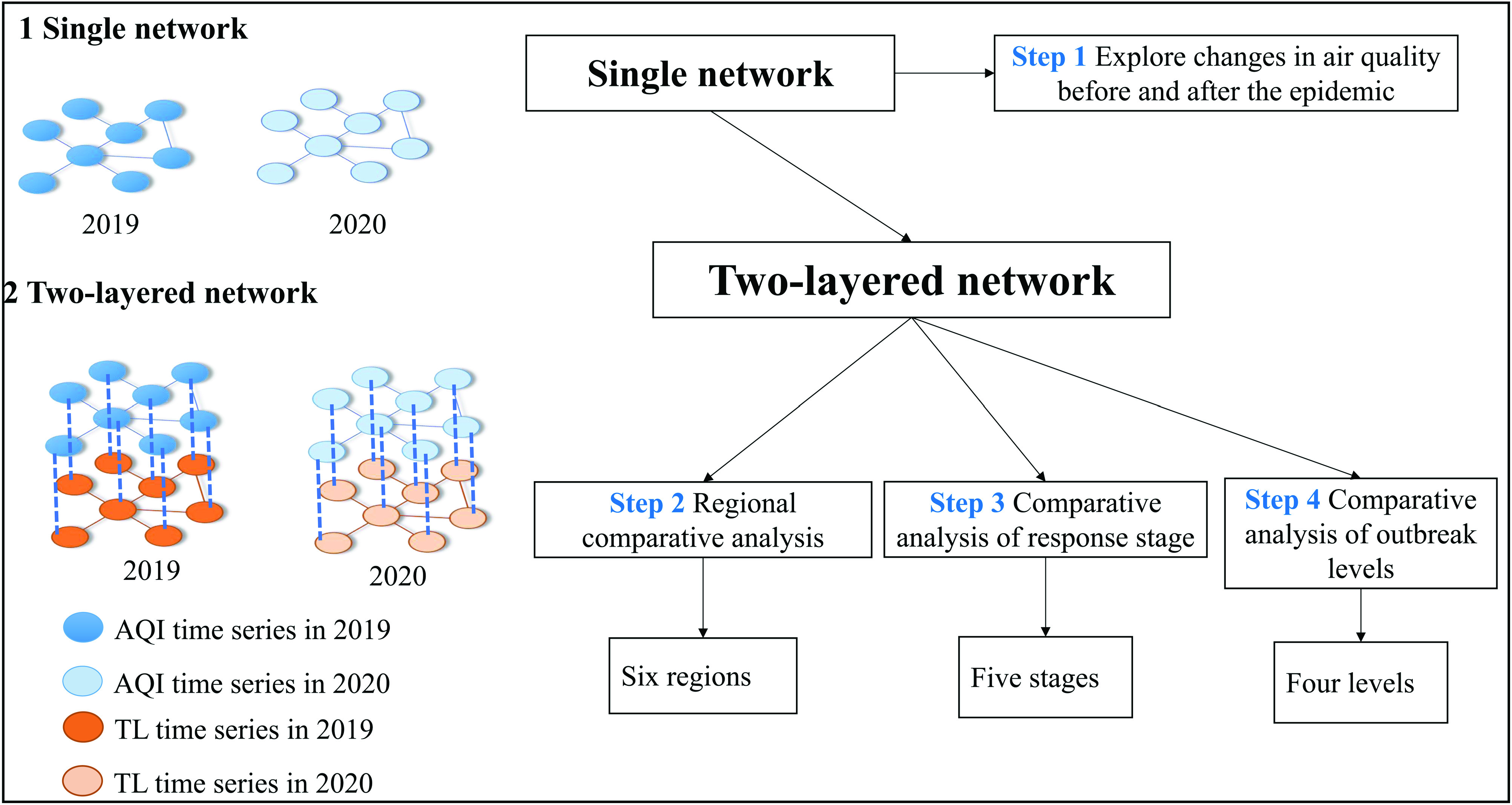
The analysis framework.

In this study, we use two types of networks: single and two-layered networks and four steps to explore the influence of traffic on air pollution during the pandemic as shown in Fig. 1. Specifically, we take the city as a node and first construct a single-layer network of air quality in 2019 and 2020 to compare and study the changes in air quality during the epidemic. Then, some multi-layer networks between the TL and the AQI are constructed to explore the impact of the epidemic on air pollution, specifically, the impact of different regions (six regions), different time stages (five stages), and outbreak levels (four levels) of the development of the epidemic.

## IV. RESULTS AND DISCUSSION

We present the main results of the correlated multi-layered networks composed of the TI and AQI as described above.

### A. The change in air pollution caused by the pandemic

**FIG. 2.**
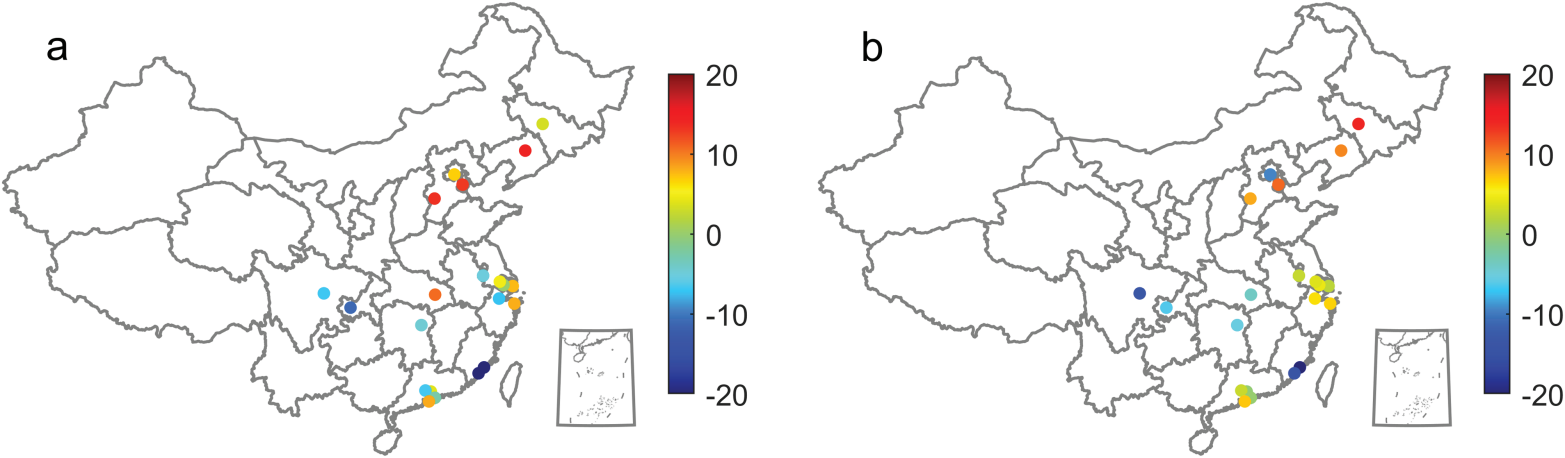
The maps of difference of weighted in-degrees (outgoing from the AQI nodes) between 2019 and 2020 (a) and the maps of difference of weighted out-degrees (incoming to the AQI nodes) between 2019 and 2020 (b).

We explore the change in air pollution caused by the pandemic. The influence of the target city’s air pollution on other cities is quantified by the weighted degrees associated with the total weights of the significant interlinks from other city nodes, which are presented in Fig. 2. A higher weighted in-degree (WID) indicates that target cities receive haze from other cities, whereas a higher weighted out-degree denotes a greater transport strength from target cities to other cities.

We find that the values of the weighted in-degrees in the Beijing–Tianjin–Hebei (BTH) and Yangtze River Delta (YRD) regions were higher in 2019. Compared with the pre-epidemic (2019) values, the values of Beijing, Tianjin, Shijiazhuang, and Changchun in North China decrease, especially in Beijing. In the YRD region, the values of Nanjing, Hangzhou, and Suzhou increase and those of other cities remain unchanged. The value of Wuhan decreases, while that of Changsha City in Central China (CC) remains the same. In southwest China (CCS), the value of Chengdu decreases and that of Chongqing increases slightly. For the Guangdong–Hong Kong–Macao Greater Bay Area (GHM), the values of Guangzhou, Dongguan, Shenzhen, and Zhuhai increase, while those of Quanzhou and Xiamen basically remain unchanged.

In terms of out-degree, the values of Nanjing and Hangzhou in the southwest region, Fujian Province, and the YRD region are higher. Compared with the pre-epidemic (2019) value in northern China, the value of Beijing becomes larger; the value of Changchun decreases and those of others remain basically unchanged. In the YRD, the value of Hangzhou becomes significantly smaller. In central China, the value of Wuhan becomes larger, while in the southwestern region, it remains basically unchanged. In the GHM, only the value of Guangzhou is significantly smaller.

As some large cities are affected by the pandemic, traffic restrictions are imposed as a result of the restrictions on production. The contribution of air pollution from other cities becomes greater.

Overall, we uncover that large cities such as Beijing, Tianjin, and Wuhan have larger in-degree values and lower out-degree values. This finding indicates that the air quality over these cities is less relative to the air condition in the cities around it. In contrast, air quality levels in Hangzhou and Guangzhou have a weak relationship with their surrounding cities, and, hence, they are more likely influenced by the epidemic. In addition, there are no distinct changes in cities over most of the CES and GHM regions, suggesting that air quality over these cities is less influenced by the epidemic.

### B. The influence of traffic on air pollution due to the pandemic

**FIG. 3.**
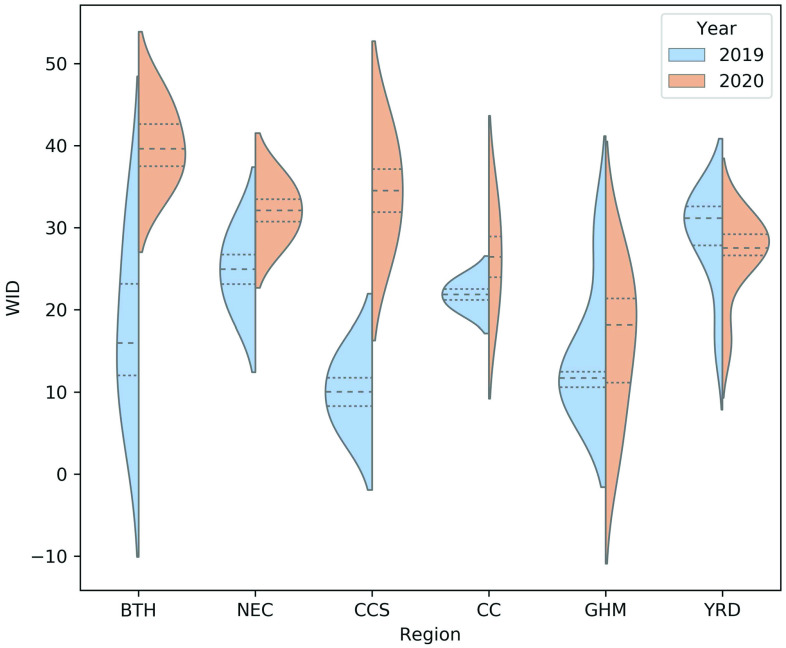
Violin plots of the weighted in-degrees (WID) that are outgoing from the TL nodes among different regions.

Based on the weighted degree index, we further study the influence of traffic on air pollution due to the pandemic in different regions by constructing a multi-layer network between the TL and the AQI. Figure 3 illustrates violin plots of the weighted in-degrees (outgoing from the TL nodes) among different regions. After comparing the years 2019 and 2020 in Fig. 2, we find that the pandemic causes significant changes in terms of the influence of traffic on air pollution. Specifically, when an epidemic occurs in the BTH region, air pollution is greatly affected by the traffic of other cities in the region (the weight in-degree value becomes higher). The influence of traffic from other cities on BTH’s air pollution changes from a dispersed one to a larger one for all cities in this region. The change in the influence of traffic on air pollution due to the pandemic in the NEC and CCS regions is similar to that in BTH. Among BTH, NEC, and CCS, the weighted in-degrees of CCS change the most compared with the situation that prevailed before the epidemic. The CC and GHM regions have larger mean values, larger maximum values, and smaller minimum values of weight in-degrees when the epidemic occurs. This suggests that some cities in these areas have closed traffic or factories for a period of time after the occurrence of the epidemic, while other cities are basically unaffected. What is more surprising is that compared to before the epidemic, for cities in the Yangtze River Delta, traffic emissions in other cities has less impact on the city's air pollution. These results indicate that either the epidemic has no major impact on the transportation, production, and life in these areas or that the recovery in these areas is faster.

Affected by the epidemic, the values over the BTH, CC, and CCS regions tend to be concentrated, suggesting that the air quality over these regions is largely related to their surrounding traffic. For the YRD and GHM regions, the values tend to be dispersed, which means that the air quality level over these regions is less influenced by traffic from other regions. Hence, their own pollution is mainly caused by the emissions and dust from mobile sources. Air pollution in the NEC region is hardly affected by the epidemic.

### C. The influence of traffic on air pollution with different stages of fighting the pandemic

**FIG. 4.**
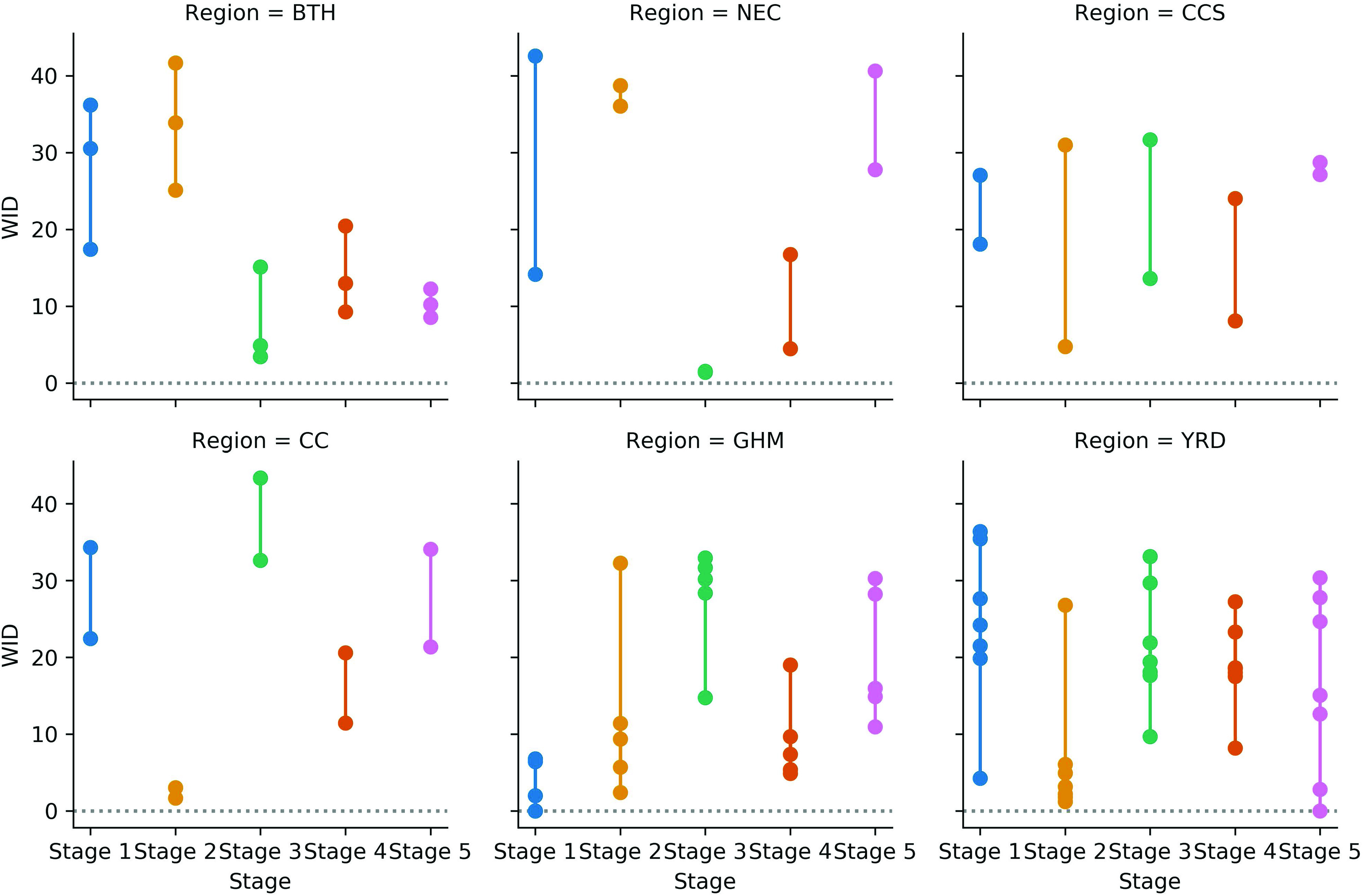
The maps of weighted in-degrees, WID (incoming to the AQI nodes and outgoing from the TL nodes) for stage I (a), II (b), III (c), IV (d), and V (e), respectively.

The epidemic situation and its control measures vary in different response stages, which will eventually lead to distinct impacts of traffic on air pollution. According to the State Council Information Office of the People's Republic of China, China's fight against COVID-19 can be divided into five stages:^49^ (I) Swift response to the public health emergency (December 27, 2019–January 19, 2020): the nationwide epidemic prevention and control plan was launched after cases were confirmed in Wuhan, as well as cases in other parts of China due to virus carriers traveling from the city; (II) Initial progress in containing the virus (January 20–February 20, 2020): The number of newly confirmed cases across the country increased rapidly, and prevention and control was extremely severe. China adopted a key measure to stop the spread of the virus by closing outbound traffic from Wuhan and Hubei; (III) Newly confirmed domestic cases on the Chinese mainland dropped to single digits (February 21–March 17, 2020): epidemic prevention and control achieved important results, people resumed work and production in an orderly manner, urban traffic resumed, and, consequently, emissions increased; (IV) Wuhan and Hubei—initial victories in a critical battle (March 18–April 28, 2020): the spread of the local epidemic in the country with Wuhan as the main battlefield was basically blocked, and control measures for outbound traffic from Wuhan and Hubei were lifted; (V) Ongoing prevention and control (since April 29, 2020): presently, the domestic epidemic situation is generally sporadic, and there are clustered epidemics caused by sporadic cases in some areas. The national epidemic prevention and control has become normalized, and traffic has returned to normal as a whole. To further study the relationship between traffic and air pollution, we classify our datasets into five stages by using time intervals of these five stages. Figure 4 illustrates the maps of weighted in-degrees for different stages.

In Fig. 4, we find that the impact of traffic in other cities on the air pollution of the BTH region reaches the maximum in stage 2 and then reaches the second peak in stage 4 in the region. For the NEC region, the impact reaches the maximum in the beginning stage and then reaches the second peak in the last stage. The impact of traffic reaches its maximum in phase 3 among the CCS and CC regions, especially in CC. For YRD, the traffic impacts in each stage are basically the same.

From a different stage perspective in Fig. 4, due to the spread of the epidemic in stage 1, travel in most cities is restricted, causing the pollution of this city to be greatly influenced by the traffic of other cities, but the GHM region is still more influenced than the other regions. Unlike in other regions where the impact is high, air pollution in the CC region is less influenced by traffic from other regions in stage 2. With the passage of time, the impact of traffic in other cities on air pollution in this city from the CC region changes from small to large in stage 3. In stage 4, the impact of traffic on air pollution in other cities is relatively small for all regions. In the stage 5, the cities whose traffic in other cities has less impact on the city's air pollution are only located in the BTH region.

Overall, the impact of traffic on air pollution in the BTH and CC regions has great fluctuations, while in the CCS, GHM, and YRD regions, the variations are much less. In Wuhan city, however, the values of the weighted in-degrees are consistent with the variations during the epidemic.

### D. The influence of traffic on air pollution with different outbreak levels in cities

**FIG. 5.**
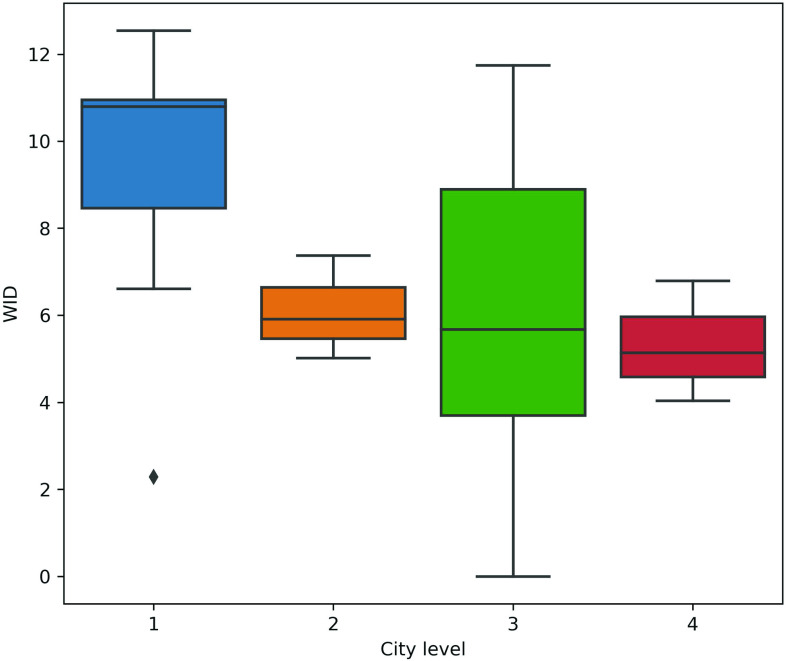
Box plots of the weighted in-degrees (WID) that are outgoing from the TL nodes among different outbreak levels in cities (city level).

Due to China’s vast land size, the development of the epidemic situation is significantly different. We need to deeply explore the relationship between traffic and air pollution in different epidemic development areas. According to the COVID-19 cumulative confirmed cases as of March 16, 2021, we classified the level of outbreak in cities (city level) into four groups as shown in Table I. Figure 5 shows the weighted in- degrees (outgoing from the TL nodes) among different outbreak levels in cities.

From the average value of view, the weighted in-degree is mainly opposed to the outbreak level in the city. It shows that the greater in-degree values, the smaller the outbreak level in the cities. When the outbreak level is high, the traffic in the target city and its surrounding areas is greatly affected by the epidemic, resulting in lesser impact of traffic in the surrounding cities on air pollution in the target city. Thus, traffic has little impact on the air quality of cities with high outbreak levels. Regarding the different outbreak levels in the cities, the changes between level 1 and level 3 are relatively large, especially for level 3.

## V. CONCLUSIONS

In this study, both single-layer and multi-layer networks have been developed to explore the influence of traffic on air pollution during the pandemic based on complex network approaches. We have found that the epidemic has less impact on air quality over Beijing, Tianjin, and Wuhan cities, while for Hangzhou and Guangzhou cities, their air quality is related to the epidemic. Compared with 2019, the air quality in the BTH, CCS, and CC regions is tied to the surrounding traffic conditions. In contrast, there are no significant changes in the YRD and GHM regions between 2019 and 2020. Furthermore, we analyzed the variations during different epidemic stages. The impact of traffic in other cities on a city's air pollution reached the maximum in stage 2. For the BTH and CC regions, the impact of traffic on air quality is large in the first two stages and then shows a decreasing trend, while for CC, a significant impact occurs in phase 3 among these regions. For other regions, there is little change in different stages. In addition, the impact over different outbreak levels is also investigated. A higher outbreak level generally has a lower in-degree value. In the case of high ranking, the traffic of the surrounding cities has less impact on the air pollution of the target city.

Compared with traditional research methods, the climate network method used in this study can explore the relationship between traffic emissions and air pollution on a larger scale, especially the long-distance impact between different cities, and more macroscopically indicate the impact of other cities**’** emissions on a city’s air pollution contribution. However, this study uses only teleconnection for network modeling and uses the index of degree for quantitative analysis. Therefore, it lacks a more detailed and refined aerodynamic transmission mechanism, making the quantification of the contribution of traffic emissions to air pollution not precise enough. The division of urban outbreak levels and epidemic development stages is relatively subjective and lacks more precise quantitative evaluation criteria. Subsequent research should choose a more scientific and effective division method. Besides providing information for guiding government policies to improve air quality levels, the development of network parameters in this work is a profitable attempt in the areas of transportation and atmospheric environment. Our results can also call attention to further research on the impact of transportation on air pollution.

## Data Availability

The data that support the findings of this study are available from the corresponding authors upon reasonable request.
